# Supplementary material for: Role and mechanism of NCAPD3 in promoting malignant behaviors in gastric cancer
Source: Front Pharmacol. 2024 Apr 22;15:1341039. doi: 10.3389/fphar.2024.1341039 (PMC11070777; doi:10.3389/fphar.2024.1341039)
Supplement: Supplementary file 11 [file DataSheet2.ZIP › GSEA/GO_CC/my_analysis.Gsea.1599461408869/GO_INTRINSIC_COMPONENT_OF_PLASMA_MEMBRANE.html]

Details for gene set GO\_INTRINSIC\_COMPONENT\_OF\_PLASMA\_MEMBRANE[GSEA]

|  || Dataset | filtered\_dataset.sample\_info.cls#WT\_versus\_NCAPD3\_MUT |
| Phenotype | sample\_info.cls#WT\_versus\_NCAPD3\_MUT |
| Upregulated in class | WT |
| GeneSet | GO\_INTRINSIC\_COMPONENT\_OF\_PLASMA\_MEMBRANE |
| Enrichment Score (ES) | 0.28899923 |
| Normalized Enrichment Score (NES) | 2.0450997 |
| Nominal p-value | 0.0 |
| FDR q-value | 0.03138242 |
| FWER p-Value | 0.075 |
Table: GSEA Results Summary

  

Fig 1: Enrichment plot: GO\_INTRINSIC\_COMPONENT\_OF\_PLASMA\_MEMBRANE      
 Profile of the Running ES Score & Positions of GeneSet Members on the Rank Ordered List

  

| SYMBOL | TITLE | RANK IN GENE LIST | RANK METRIC SCORE | RUNNING ES | CORE ENRICHMENT || 1 | 659 | BMPR2 | 16 | 1.047 | 0.0027 | Yes |
| 2 | 7052 | TGM2 | 17 | 1.045 | 0.0176 | Yes |
| 3 | 5099 | PCDH7 | 23 | 1.026 | 0.0284 | Yes |
| 4 | 54894 | RNF43 | 29 | 1.000 | 0.0388 | Yes |
| 5 | 84133 | ZNRF3 | 30 | 0.996 | 0.0531 | Yes |
| 6 | 3654 | IRAK1 | 69 | 0.856 | 0.0361 | Yes |
| 7 | 3667 | IRS1 | 83 | 0.821 | 0.0378 | Yes |
| 8 | 56110 | PCDHGA5 | 102 | 0.781 | 0.0352 | Yes |
| 9 | 56107 | PCDHGA9 | 103 | 0.781 | 0.0463 | Yes |
| 10 | 56101 | PCDHGB5 | 104 | 0.781 | 0.0575 | Yes |
| 11 | 56100 | PCDHGB6 | 105 | 0.781 | 0.0687 | Yes |
| 12 | 56113 | PCDHGA2 | 106 | 0.781 | 0.0798 | Yes |
| 13 | 56112 | PCDHGA3 | 107 | 0.781 | 0.0910 | Yes |
| 14 | 56099 | PCDHGB7 | 108 | 0.781 | 0.1022 | Yes |
| 15 | 9708 | PCDHGA8 | 109 | 0.781 | 0.1133 | Yes |
| 16 | 56105 | PCDHGA11 | 110 | 0.781 | 0.1245 | Yes |
| 17 | 56109 | PCDHGA6 | 111 | 0.781 | 0.1356 | Yes |
| 18 | 56098 | PCDHGC4 | 112 | 0.781 | 0.1468 | Yes |
| 19 | 56097 | PCDHGC5 | 113 | 0.781 | 0.1580 | Yes |
| 20 | 56111 | PCDHGA4 | 114 | 0.781 | 0.1691 | Yes |
| 21 | 56104 | PCDHGB1 | 115 | 0.781 | 0.1803 | Yes |
| 22 | 56106 | PCDHGA10 | 116 | 0.781 | 0.1915 | Yes |
| 23 | 56114 | PCDHGA1 | 117 | 0.781 | 0.2026 | Yes |
| 24 | 5098 | PCDHGC3 | 118 | 0.781 | 0.2138 | Yes |
| 25 | 26025 | PCDHGA12 | 119 | 0.781 | 0.2250 | Yes |
| 26 | 56103 | PCDHGB2 | 120 | 0.781 | 0.2361 | Yes |
| 27 | 56108 | PCDHGA7 | 121 | 0.781 | 0.2473 | Yes |
| 28 | 8641 | PCDHGB4 | 122 | 0.781 | 0.2585 | Yes |
| 29 | 56102 | PCDHGB3 | 123 | 0.781 | 0.2696 | Yes |
| 30 | 8549 | LGR5 | 148 | 0.739 | 0.2617 | Yes |
| 31 | 55714 | TENM3 | 149 | 0.736 | 0.2722 | Yes |
| 32 | 3770 | KCNJ14 | 157 | 0.721 | 0.2772 | Yes |
| 33 | 10161 | LPAR6 | 163 | 0.711 | 0.2835 | Yes |
| 34 | 134957 | STXBP5 | 170 | 0.709 | 0.2890 | Yes |
| 35 | 145389 | SLC38A6 | 202 | 0.673 | 0.2748 | No |
| 36 | 81619 | TSPAN14 | 212 | 0.666 | 0.2774 | No |
| 37 | 6573 | SLC19A1 | 231 | 0.650 | 0.2728 | No |
| 38 | 7220 | TRPC1 | 328 | 0.576 | 0.2073 | No |
| 39 | 960 | CD44 | 339 | 0.573 | 0.2078 | No |
| 40 | 10818 | FRS2 | 363 | 0.558 | 0.1981 | No |
| 41 | 1080 | CFTR | 366 | 0.557 | 0.2045 | No |
| 42 | 1183 | CLCN4 | 373 | 0.551 | 0.2078 | No |
| 43 | 6507 | SLC1A3 | 464 | 0.496 | 0.1457 | No |
| 44 | 4987 | OPRL1 | 477 | 0.487 | 0.1434 | No |
| 45 | 5245 | PHB | 481 | 0.486 | 0.1480 | No |
| 46 | 81031 | SLC2A10 | 539 | 0.453 | 0.1107 | No |
| 47 | 7099 | TLR4 | 541 | 0.451 | 0.1164 | No |
| 48 | 10280 | SIGMAR1 | 655 | 0.399 | 0.0352 | No |
| 49 | 51283 | BFAR | 676 | 0.389 | 0.0254 | No |
| 50 | 5311 | PKD2 | 708 | 0.372 | 0.0069 | No |
| 51 | 23767 | FLRT3 | 781 | 0.333 | -0.0437 | No |
| 52 | 57181 | SLC39A10 | 811 | 0.313 | -0.0615 | No |
| 53 | 7037 | TFRC | 824 | 0.303 | -0.0664 | No |
| 54 | 6558 | SLC12A2 | 839 | 0.276 | -0.0732 | No |
| 55 | 4071 | TM4SF1 | 851 | -0.251 | -0.0781 | No |
| 56 | 3673 | ITGA2 | 858 | -0.264 | -0.0789 | No |
| 57 | 6385 | SDC4 | 871 | -0.282 | -0.0841 | No |
| 58 | 6515 | SLC2A3 | 873 | -0.283 | -0.0809 | No |
| 59 | 2150 | F2RL1 | 887 | -0.304 | -0.0865 | No |
| 60 | 3691 | ITGB4 | 926 | -0.352 | -0.1107 | No |
| 61 | 3949 | LDLR | 929 | -0.353 | -0.1072 | No |
| 62 | 4973 | OLR1 | 944 | -0.366 | -0.1127 | No |
| 63 | 9052 | GPRC5A | 951 | -0.369 | -0.1121 | No |
| 64 | 5329 | PLAUR | 952 | -0.373 | -0.1067 | No |
| 65 | 4864 | NPC1 | 964 | -0.384 | -0.1097 | No |
| 66 | 5621 | PRNP | 970 | -0.387 | -0.1080 | No |
| 67 | 9180 | OSMR | 981 | -0.394 | -0.1101 | No |
| 68 | 9308 | CD83 | 1025 | -0.423 | -0.1371 | No |
| 69 | 7871 | SLMAP | 1038 | -0.435 | -0.1401 | No |
| 70 | 966 | CD59 | 1040 | -0.436 | -0.1346 | No |
| 71 | 7132 | TNFRSF1A | 1055 | -0.449 | -0.1390 | No |
| 72 | 1308 | COL17A1 | 1065 | -0.455 | -0.1394 | No |
| 73 | 2523 | FUT1 | 1068 | -0.456 | -0.1344 | No |
| 74 | 5818 | PVRL1 | 1073 | -0.458 | -0.1310 | No |
| 75 | 9076 | CLDN1 | 1084 | -0.463 | -0.1320 | No |
| 76 | 857 | CAV1 | 1098 | -0.475 | -0.1352 | No |
| 77 | 3556 | IL1RAP | 1099 | -0.476 | -0.1284 | No |
| 78 | 10098 | TSPAN5 | 1112 | -0.485 | -0.1307 | No |
| 79 | 2706 | GJB2 | 1115 | -0.490 | -0.1252 | No |
| 80 | 9122 | SLC16A4 | 1125 | -0.498 | -0.1251 | No |
| 81 | 3038 | HAS3 | 1130 | -0.501 | -0.1210 | No |
| 82 | 5097 | PCDH1 | 1132 | -0.502 | -0.1146 | No |
| 83 | 5359 | PLSCR1 | 1146 | -0.511 | -0.1173 | No |
| 84 | 9962 | SLC23A2 | 1169 | -0.537 | -0.1265 | No |
| 85 | 2825 | GPR1 | 1171 | -0.538 | -0.1196 | No |
| 86 | 54407 | SLC38A2 | 1182 | -0.553 | -0.1194 | No |
| 87 | 2152 | F3 | 1188 | -0.559 | -0.1152 | No |
| 88 | 5795 | PTPRJ | 1210 | -0.586 | -0.1230 | No |
| 89 | 153 | ADRB1 | 1221 | -0.595 | -0.1222 | No |
| 90 | 2043 | EPHA4 | 1228 | -0.602 | -0.1182 | No |
| 91 | 50856 | CLEC4A | 1249 | -0.630 | -0.1246 | No |
| 92 | 1956 | EGFR | 1266 | -0.657 | -0.1275 | No |
| 93 | 80328 | ULBP2 | 1277 | -0.664 | -0.1257 | No |
| 94 | 8501 | SLC43A1 | 1296 | -0.690 | -0.1296 | No |
| 95 | 3084 | NRG1 | 1320 | -0.739 | -0.1368 | No |
| 96 | 1839 | HBEGF | 1328 | -0.751 | -0.1314 | No |
| 97 | 6509 | SLC1A4 | 1330 | -0.752 | -0.1214 | No |
| 98 | 7779 | SLC30A1 | 1332 | -0.755 | -0.1114 | No |
| 99 | 27076 | LYPD3 | 1348 | -0.790 | -0.1117 | No |
| 100 | 4233 | MET | 1358 | -0.821 | -0.1069 | No |
| 101 | 79465 | ULBP3 | 1366 | -0.847 | -0.1001 | No |
| 102 | 5794 | PTPRH | 1368 | -0.854 | -0.0887 | No |
| 103 | 9966 | TNFSF15 | 1376 | -0.900 | -0.0812 | No |
| 104 | 9990 | SLC12A6 | 1379 | -0.922 | -0.0696 | No |
| 105 | 5156 | PDGFRA | 1383 | -0.932 | -0.0586 | No |
| 106 | 4919 | ROR1 | 1385 | -0.954 | -0.0457 | No |
| 107 | 6541 | SLC7A1 | 1388 | -0.981 | -0.0333 | No |
| 108 | 2040 | STOM | 1389 | -0.988 | -0.0191 | No |
| 109 | 493 | ATP2B4 | 1401 | -1.166 | -0.0109 | No |
| 110 | 6519 | SLC3A1 | 1404 | -1.195 | 0.0046 | No |
Table: GSEA details [plain text format]

  

Fig 2: GO\_INTRINSIC\_COMPONENT\_OF\_PLASMA\_MEMBRANE      
 Blue-Pink O' Gram in the Space of the Analyzed GeneSet

  

Fig 3: GO\_INTRINSIC\_COMPONENT\_OF\_PLASMA\_MEMBRANE: Random ES distribution      
 Gene set null distribution of ES for **GO\_INTRINSIC\_COMPONENT\_OF\_PLASMA\_MEMBRANE**
